# Academic Resilience and its Relationship With Emotional Intelligence and Stress Among University Students: A Three‐Country Survey

**DOI:** 10.1002/brb3.70497

**Published:** 2025-04-21

**Authors:** Hanan Abdelrahman, Mohammad Al Qadire, Suha Ballout, Mohammad Rababa, Esther Nana Kwaning, Hamada Zehry

**Affiliations:** ^1^ Manning College of Nursing and Health Sciences University of Massachusetts Boston Boston Massachusetts USA; ^2^ Faculty of Nursing Suez Canal University Ismailia Egypt; ^3^ Adult Health Department, Faculty of Nursing Al Al‐Bayt University Mafraq Jordan; ^4^ Department of Adult Health Nursing, Faculty of Nursing Jordan University of Science and Technology Irbid Jordan; ^5^ College of Nursing Sulaiman AlRajhi University Al Bukayriah Saudi Arabia; ^6^ Faculty of Medicine Horus University‐ Egypt Damietta City Egypt

**Keywords:** academic resilience, cross‐cultural study, emotional intelligence, middle east, perceived stress, university students

## Abstract

**Background:**

In Mediterranean countries, resilience among university students remains underexplored, despite its critical role in managing academic and personal stressors. Emotional intelligence (EI) and perceived stress are known to influence resilience but require further investigation in culturally diverse settings.

**Aim:**

To explore the relationships between academic resilience, EI, and perceived stress among university students in three Mediterranean countries characterized by diverse academic and cultural systems.

**Sample:**

1833 undergraduate students from 12 universities in Egypt, Jordan, and Oman.

**Methods:**

A cross‐sectional correlational design was employed. Participants completed the academic resilience scale (ARS‐30), Schutte self‐report emotional intelligence test (SSEIT), and perceived stress scale (PSS). Multiple linear regression was used to identify predictors of academic resilience.

**Results:**

The mean resilience score was 67.4 (SD = 18.4). EI positively predicted resilience (B = 0.57, β = 0.66, p < 0.001), whereas perceived stress was negatively associated (B = ‐0.48, β = 0.15, p < 0.001). Other significant predictors included good health, advanced academic years, GPA, nationality, and participation in stress‐management training. Variables, such as gender, age, and field of specialty, did not significantly influence resilience. The model explained 54% of the variance in resilience (R^2^ = 0.54, p < 0.001).

**Conclusions:**

This study provides novel insights into the predictors of resilience in culturally diverse Mediterranean contexts. Emotional intelligence and stress management are critical for enhancing academic resilience. Universities should integrate EI training, stress reduction programs, and resilience‐building initiatives into their curricula. Future research should explore longitudinal trends and culturally tailored interventions to promote resilience.

## Graphical abstract

This cross‐cultural study of 1833 students from Egypt, Jordan, and Oman found that emotional intelligence significantly enhances academic resilience, while perceived stress reduces it. Health, academic progress, GPA, and stress management training also play key roles, highlighting the need to integrate EI and stress‐reduction strategies into university curricula.

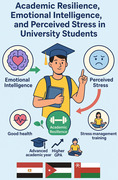

## 1 Background

University students navigate a complex landscape of academic, social, and personal challenges, which can significantly impact their mental health and academic performance. The transition to higher education, coupled with increasing academic demands, financial pressures, and social adjustments, often makes students vulnerable to stress and burnout (Pascoe et al. 2020). Academic resilience refers to a student's capacity to effectively deal with academic setbacks, stress, and adversity and still achieve successful outcomes in their studies. It is considered to be a key component of student success in higher education (Martin and Marsh 2006). Cassidy (2016) defined academic resilience as the ability to sustain high levels of motivation and performance, despite encountering academic challenges. This construct includes the use of cognitive, emotional, and behavioral strategies that help students manage academic pressures, limited resources, and personal difficulties, while maintaining engagement and goal‐directed behavior (Martin 2013). According to Morales (2008), resilient students often demonstrate self‐regulation, persistence, and a positive academic self‐concept that buffers the effects of adversity and promotes achievement. Recent studies have emphasized that academic resilience not only contributes to academic success, but also plays a protective role in students’ mental health and overall well‐being (Demir 2023; Yang and Wang 2022).

Resilient students demonstrate a heightened capacity to adapt to stressors and succeed, even in adverse circumstances, highlighting their critical role in fostering persistence and well‐being (Năstasă et al. 2022; Ononye, Ndudi, Bereprebofa, and Maduemezia 2022; Radhamani and Kalaivani 2021). Similarly, emotional intelligence (EI), which encompasses the ability to perceive, understand, regulate, and use emotions effectively, is a pivotal factor in academic success (Mayer et al. 2004). It plays a critical role in students’ abilities to navigate academic demands and social environments. Students with high emotional intelligence often demonstrate superior stress management skills, stronger interpersonal relationships, and greater adaptability (Goleman 2005; Schutte et al. 1998). These emotional competencies not only enhance academic performance but also promote psychological well‐being, resilience, and motivation (Hwang and Kim 2023; Trigueros et al. 2020). Moreover, emotional intelligence enables students to process challenges more constructively, reducing academic anxiety, and fostering sustained engagement with learning (Petrides 2011).

Emotional intelligence facilitates the development of resilience, further enabling students to navigate challenges effectively (Ahmed et al. 2018; Beri and Kumar 2018; Wei and Song 2024)​​​. This interplay between emotional intelligence and resilience underscores their importance in academic contexts (Trigueros et al. 2020). Furthermore, emotional intelligence mediated the relationship between social support and academic resilience among Turkish university students (Kökçam et al. 2022).

Perceived stress refers to an individual's subjective evaluation of how unpredictable, uncontrollable, or overwhelming they find their life circumstances, particularly when they feel that external demands exceed their coping resources (Cohen et al. 1983). Unlike objective stressors, perceived stress centers on personal appraisal and emotional responses, making it a crucial factor in psychological outcomes (Lazarus 1984; Slimmen, Timmermans, Mikolajczak‐Degrauwe, and Oenema 2022). In the academic context, high levels of perceived stress are consistently associated with reduced academic performance, diminished well‐being, and lower levels of resilience (Ribeiro et al. 2018; Slimmen et al. 2022). University students are particularly vulnerable, as they face multiple pressures related to academic workload, financial concerns, social expectations, and life transitions (Pascoe et al. 2020). For instance, university students often experience moderate to high‐stress levels, with educational and environmental stressors being the most significant contributors (Alkhawaldeh et al. 2023).

Resilience is protective in university students, showing that higher resilience levels correlate with better psychological well‐being, even under significant stress (Zaheer and Khan 2022). Similarly, Ribeiro et al. (2018) noted that stress negatively affects students' quality of life and academic outcomes, emphasizing the need for targeted stress management programs. Emotional intelligence enhances resilience by helping students regulate their emotional responses and maintain a positive outlook (Beri and Kumar 2018; Wei and Song 2024). Resilience mitigates the impact of stress on academic outcomes by promoting persistence and adaptability. This dynamic relationship highlights the importance of developing resilience and emotional intelligence to support students in managing their educational demands (Trigueros et al. 2020).

Despite the growing body of research, more comprehensive studies are needed to explore the interplay between academic resilience, emotional intelligence, perceived stress, and other predictors across diverse cultural settings. While previous studies have confirmed the relationships among these variables, they often fail to account for cultural and contextual differences, particularly in Mediterranean countries. This study addresses this gap by examining these constructs within the unique socio‐cultural contexts of Egypt, Jordan, and Oman. These countries represent a diverse mix of academic and societal environments, which can influence resilience and its predictors in distinct ways. For example, strong familial bonds and expectations in Egypt often shape students’ academic motivations and coping strategies. Jordanian students may face unique stressors related to limited resources and competitive academic environments, while Omani students often benefit from robust governmental support programs to foster educational success. These societal and academic factors create a rich context for exploring how resilience is developed and influenced.

This study is justified by the increasing global emphasis on student mental health and academic success, particularly in the wake of the COVID‐19 pandemic, which exacerbated stress and mental health challenges among university students (Sahu 2020). Understanding the factors that promote academic resilience is essential for developing targeted interventions to help students overcome adversity and achieve their educational goals. Focusing on Mediterranean countries, this research provides new insights into how cultural, academic, and personal factors shape resilience, offering actionable recommendations for educators and policymakers. These recommendations will be elaborated further in the discussion and conclusion sections, addressing specific interventions and strategies tailored to the unique contexts of these regions. Therefore, this study aimed to assess academic resilience and its association with emotional intelligence and perceived stress among university students across three Mediterranean countries.

## 2 Hypotheses

1. **H1**: Emotional intelligence is a positive predictor of academic resilience among university students.
2. **H2**: Perceived stress is a negative predictor of academic resilience among university students.
3. **H3**: Demographic and academic variables (e.g., Grade Point Average (GPA), academic year, nationality, health status, participation in stress management training) significantly predict academic resilience.

## 3 Materials and Methods

### 3.1 Design

A cross‐sectional correlational design explored the relationships among academic resilience, emotional intelligence, and perceived stress in diverse cultural contexts.

### 3.2 Settings

This study was conducted across 12 universities in three countries: six in Egypt, four in Jordan, and two in Oman. The participating institutions represented diverse educational settings, comprising eight governmental and four private universities. These universities were carefully selected to ensure representation in various academic specialties, including medical and non‐medical disciplines. These universities were carefully selected to ensure representation in various academic specialties, including medical and non‐medical disciplines, providing a diverse context for understanding resilience predictors. The selection also considered geographical and institutional diversity to capture various experiences.

### 3.3 Sample and Sampling

The study comprised 1833 undergraduate students enrolled in various colleges who expressed a willingness to participate. To maintain a focused and relevant sample, students enrolled in postgraduate or Ph.D. programs and those who declined to participate were excluded. Participants were selected using a convenience sampling technique, which, while practical for reaching a large number of respondents, may limit the generalizability of findings. Demographic data were carefully analyzed to address this limitation and ensure that key subgroups, such as gender, academic fields, and socio‐economic status, were proportionally represented. Efforts were made to enhance representativeness by targeting diverse academic fields and student demographics through strategic outreach to departments with varying student compositions, including STEM, humanities, and professional programs. Additionally, specific campaigns encouraged participation from underrepresented groups to ensure broader inclusivity.

#### 3.3.1 Sample Size Calculation

The total estimated population of nursing students across the three participating countries was approximately 50,000, comprising 20,000 students in Egypt and 10,000 students each in Jordan and Oman. The required sample size was calculated using G*Power 3.1 software for multiple linear regression analysis (fixed model, R^2^ deviation from zero), with the following parameters: small effect size (f^2^ = 0.02), alpha level of 0.05, desired statistical power of 0.80, and 25 predictors. Based on these parameters, the minimum required sample size was 1682 participants. To increase statistical power and account for potential non‐response or incomplete data, the sample size was increased by 151 participants, resulting in a final sample size of 1833. The participants were recruited proportionally from each country according to the estimated student population.

### 3.4 Measurements

The demographics questionnaire was used to collect the key characteristics of the study population, providing a comprehensive profile of the participants. The questionnaire included variables such as sex, nationality, academic field, academic year, GPA, average weekly study hours, part‐time job status, participation in extracurricular activities, health status, prior training on stress management, frequency of stress management practices, and a support system.

The academic resilience scale (ARS‐30) was developed to evaluate academic resilience by measuring individuals’ cognitive, emotional, and behavioral responses to academic challenges (Cassidy 2016). The scale consists of 30 items scored on a 5‐point Likert scale ranging from “unlikely” (1) to “likely” (5). It measures three dimensions: adverse impact and emotional reaction, reflection and adaptive help‐seeking, and perseverance (Cassidy 2016). The scale has demonstrated high reliability and internal consistency. The alpha coefficients were 0.96 for perseverance, 0.84 for reflection and adaptive help‐seeking, and 0.86 for adverse emotional reaction and negative affect, with an overall alpha coefficient of 0.93 (Ramezanpour et al. 2019). The scale's language and cultural relevance were reviewed for appropriateness in the study's context by engaging bilingual experts who assessed the translations and cultural adaptability of the items. Feedback was incorporated to ensure clarity and relevance to the diverse populations included in the study.

The Schutte self‐report emotional intelligence test (SSEIT) was developed to measure emotional intelligence using 33 self‐report items (Schutte et al. 1998). The scale comprises four subscales: emotional perception (10 items), self‐management of emotions (nine items), management of others’ emotions (eight items), and utilization of emotions (six items) (Hussein, Acquah, and Musah 2019). Participants responded using a 5‐point Likert scale ranging from “strongly disagree” (1) to “strongly agree” (5). The total scores range from 33 to 165, with higher scores indicating greater emotional intelligence. Scores below 111 and above 137 are considered extremely low and high, respectively, while the average emotional intelligence score is 124 (Caboral‐Stevens, Sedhom, and Rosario‐Sim 2016). SSEIT demonstrates excellent internal consistency with an overall alpha coefficient of 0.96 (Weerasinghe et al. 2023).

The perceived stress scale (PSS) is a widely recognized tool for assessing psychological stress (Huang et al. 2020). The 10‐item scale uses a 5‐point Likert response format, ranging from (0) “never” to (4) “very often”. Items were categorized into six negative components (items 1, 2, 3, 8, 11, and 14) and four positive components (items 6, 7, 9, and 10) (Huang et al. 2020). Total scores ranged from 0 to 40, with higher scores indicating greater perceived stress (Ranasinghe, Wathurapatha, Mathangasinghe, and Ponnamperuma 2017). The scale has demonstrated internal consistency with a Cronbach's alpha coefficient of 0.74 (Williamson et al. 1988). A pilot test confirmed its appropriateness for the target population by recruiting a small sample of participant's representative of the study demographics. Feedback from the pilot was analyzed to identify potential issues with clarity, cultural relevance, or item interpretation, and adjustments were made to ensure the survey was accessible and contextually appropriate for diverse populations.

### 3.5 Data Collection Procedure

Data for this study were collected through an online survey designed using Google Forms, ensuring widespread accessibility and participation across the three countries. Efforts were made to minimize digital access barriers by providing participants with multiple reminders and alternative submission options if technical difficulties arose. The survey was distributed to students from 12 universities across the three countries. Approval for conducting the survey was obtained from the administrative authorities of each university before initiating the data‐collection process. After obtaining approval, the survey link was shared with the students through their university email portals to ensure secure and direct access. The first page of the survey included a detailed explanation of the study's purpose, objectives, and participation requirements. Participants were informed that completing the survey, which required approximately 20–25 min, would be considered as providing their consent to participate in the study. The information also assured participants of the voluntary nature of the survey, the confidentiality of their responses, and the anonymity of their participation. The data collection period spanned eight weeks; reminder emails were sent every two weeks via the student email portals to enhance the response rate. Additional measures, such as personalized reminders, were implemented to improve participation rates. These reminders effectively increased engagement, with participation rates rising by approximately 15% after implementation, ensuring a more robust and representative dataset. These reminders effectively increased engagement, with participation rates rising by approximately 15% after implementation, ensuring a more robust and representative dataset.

### 3.6 Ethical Considerations

Approval for this study was obtained from appropriate Research Ethics Committees and relevant university administrations in the three countries involved, ensuring compliance with international ethical research standards. Ethical principles, including voluntary participation, confidentiality, and informed consent, were rigorously upheld. Participants received clear and detailed information about the study's purpose, procedures, and rights. Consent was implied by the participants’ voluntary completion of the online survey, as explained on the first page of the questionnaire. All data were stored on a password‐protected computer, with access restricted exclusively to research team members. Cultural sensitivity and participant privacy were prioritized throughout the study, particularly given the cross‐cultural context. Specific actions included consulting with local academic advisors to ensure that survey questions were culturally appropriate and non‐offensive, translating and back‐translating materials to maintain linguistic accuracy, and conducting sensitivity training for researchers involved in the data collection. These steps ensured that the study respected the unique cultural dynamics of the populations concerned.

### 3.7 Data Analysis

The data were analyzed using IBM SPSS statistical software version 29.0. A detailed data cleaning process was performed before analysis to ensure accuracy and consistency. The data cleaning involved checking for missing values, outliers, and duplicate entries. Missing data were handled using appropriate imputation methods to minimize bias and maintain dataset integrity. Outliers were examined to determine their potential impact on regression results. Assumptions for normality and homoscedasticity were evaluated to ensure the validity of subsequent analyses. Descriptive statistics provided insights into sample characteristics and highlighted potential trends in emotional intelligence, perceived stress, and academic resilience. Inferential statistical techniques were employed following the descriptive analysis to explore relationships further and identify predictors.

Inferential analysis was conducted to identify significant predictors of academic resilience, aligning with the study's aim to explore the interplay of emotional intelligence, stress, and demographic factors. Multiple linear regression analysis was performed using an enter method, including emotional intelligence, perceived stress, and demographic variables as predictors. Before conducting regression analysis, assumptions such as multicollinearity, linearity, and normality of residuals were tested. Variance Inflation Factor (VIF) was used to assess multicollinearity, ensuring the reliability of predictors. In addition to p‐values, effect sizes, and 95% confidence intervals were reported to provide a more comprehensive understanding of the predictors' impact.

## 4 Results

### 4.1 Sample Characteristic

The average age of participants was 20.7 years (SD = 2.4), with a mean GPA of 3.1 (SD = 0.6). Participants spent 22.8 h per week studying (SD = 16.5). The majority were Egyptian (57.7%), female (70.8%), and enrolled in non‐medical disciplines (54.1%). Most participants (84.6%) did not work part‐time jobs, and 83.9% had not received stress management training. Notably, 70.3% of students reported having a support system, and 51.8% participated in extracurricular activities. As shown in Table 1, fifth‐year students represented the most prominent academic group (24.9%), potentially reflecting the resilience required to persist in higher education. These characteristics provide an important context for understanding emotional intelligence, perceived stress, and academic resilience in the sample population.

**TABLE 1:** Demographic characteristics.

Characteristic	Category	Frequency (%)	Mean (SD)
Age (Year)			20.7 (2.4)
GPA			3.1 (0.6)
Studying hours (Hrs.)			22.8 (16.5)
Nationality	Egypt	1058 (57.7)	
	Jordan	443 (24.2)	
	Oman	332 (18.1)	
Gender	Female	1298 (70.8)	
	Male	535(29.2)	
Academic year	1st year	383 (20.9)	
	2nd year	424 (23.1)	
	3rd year	227 (12.4)	
	4th year	343 (18.7)	
	5th year	456 (24.9)	
Part‐time job	No	1550 (84.6)	
	Yes	296 (15.4)	
Training on stress management	No	1537 (83.9)	
	Yes	296 (16.1)	
Academic field	Non‐medical	992 (54.1)	
	Medical and health sciences	841 (45.9)	
Health status	Poor or fair	281(15.3)	
	Good or excellent	1552 (84.7)	
Extracurricular activity	Rarely	884 (48.2)	
	Regular	949 (51.8)	
Stress management	Rarely	742 (40.4)	
	Regular	1091 (59.6)	
Having a support system?	No	543 (29.7)	
	Yes	1290 (70.3)	

### 4.2 Academic Resilience and its Predictors

The mean score for academic resilience was 67.4 (SD = 18.4), with scores ranging from 30 to 139. Emotional intelligence had a mean score of 121.9 (SD = 20.5), and perceived stress averaged 21.4 (SD = 5.9), indicating moderate stress levels.

Multiple linear regression analysis revealed that the model explained 54% of the variance in resilience (R^2^ = 0.54, adjusted R^2^ = 0.53, p < 0.001). Emotional intelligence (β = 0.66, p < 0.001) and perceived stress (β = ‐0.15, p < 0.001) were the strongest predictors of resilience, emphasizing the importance of emotional regulation and stress management in fostering adaptability. Students with good health (β = 0.06, p = 0.001), a support system (β = 0.06, p = 0.003), and stress management training (β = 0.04, p = 0.030) also demonstrated higher resilience.

Resilience increased progressively with the academic year, with fifth‐year students showing the greatest resilience (β = 0.14, p < 0.001). GPA and nationality were additional significant predictors, with Jordanian (β = 0.07, p = 0.006) and Omani students (β = 0.06, p = 0.014) exhibiting greater resilience than Egyptian students.

Non‐significant variables, including sex, age, part‐time work, extracurricular activities, and field of specialty, suggest that personal and contextual factors play a larger role than academic variables in influencing resilience. These findings highlight the need for targeted interventions, such as emotional intelligence training and stress management programs, to enhance resilience among university students. Table 2 presents full details of the regression model.

**TABLE 2:** Predictors of academic reliance among university students.

Variable	Unstandardized coefficients		Standardized coefficients	t	P	95 % CI for B
	B	Std. error	Beta			
(Constant)	132.6	5.03		26.36	< 0.001	122.79 to 142.53
Emotional intelligence	0.57	0.02	0.66	32.83	< 0.001	0.54 to 0.60
Perceived stress level	−0.48	0.06	0.15	7.16	< 0.001	−0.35 to ‐0.61
Specialty (medical with reference to non‐medical)	0.25	0.86	0.01	0.29	0.766	−1.43 to 1.95
Health status (good with reference to poor)	3.64	1.10	0.06	3.29	0.001	1.47 to 5.81
Studying hours (Hrs.)	0.735	0.78	0.02	0.94	0.347	−.797 to 2.268
Stress management (regular with reference to rarely)	0.837	0.78	0.02	1.06	0.288	−2.38 to 0.70
Support system (yes with reference to no)	2.39	0.80	0.06	2.96	0.003	0.81to 3.97
Academic year						
Second year (with reference to first year)	3.15	1.49	0.07	2.11	0.035	0.22 to 6.07
Third year (with reference to first year)	4.35	1.62	0.08	2.67	0.007	1.16 to 7.54
Fourth year (with reference to first year)	4.26	1.66	0.08	2.55	0.011	0.99 to 7.53
Fifth year (with reference to first year)	5.60	1.59	0.14	3.52	< 0.001	2.48 to 8.72
Gender (male with reference to female)	−0.937	0.82	−0.02	−1.14	0.252	−2.54 to 0.66
Age (years)	0.01	0.18	0.01	0.04	0.966	−0.34 to 0.361
GPA (4 point)	1.32	0.63	0.04	2.08	0.038	0.07 to 2.56
Part time work (yes with reference to no)	0.284	1.08	0.01	0.26	0.794	−1.84 to 2.41
Extracurricular activity (regular with reference to rarely)	−0.01	1.02	−0.01	−0.65	0.515	−0.05 to 0.02
Jordanian with reference to Egyptian	3.01	1.09	0.07	2.75	0.006	0.86 to 5.15
Omani with reference to Egyptian	2.62	1.066	0.06	2.46	0.014	0.53 to 4.71
Training on stress management	2.15	0.98	0.04	2.18	0.30	0.21 to 4.09

P significant at < 0.05, R^2^ = 54%, Adjusted R^2^ = 53%.

## 5 Discussion

This study contributes to the growing literature on the association between EI, perceived stress, and academic resilience among university students. This study provides novel insights into resilience among students in Mediterranean countries, particularly highlighting how cultural and systemic factors, such as family support structures and educational policies, influence resilience levels. The mean resilience score observed in this study (67.4, SD = 18.4) aligns with prior research that has consistently identified resilience as a critical factor in helping students adapt to academic challenges and thrive in demanding environments (Smith et al. 2008). This study's mean EI score of 121.9 (SD = 20.5) aligns with previous research, highlighting the positive relationship between emotional intelligence and resilience. For instance, individuals with higher EI demonstrate enhanced resilience because of their ability to regulate emotions and manage stress effectively (Hwang and Kim 2023; Trigueros et al. 2020). These findings are consistent with the work of Mayer et al. (2004), which highlighted that EI fosters psychological well‐being and adaptability in the face of adversity. The ability to understand and manage emotions has been shown to reduce stress and increase confidence, which are the key resilience factors. Moreover, this study's positive correlation between EI and resilience reinforces previous findings, suggesting that EI training could play a significant role in student resilience‐building interventions.

In the academic context, higher EI equips students with the capacity to manage academic stress, maintain motivation, and seek social support when needed. These attributes not only enhance resilience but also contribute to improving academic outcomes. For example, university students with higher EI reported lower levels of academic stress and exam anxiety, which are often detrimental to their resilience (Trigueros et al. 2020). Similarly, emotional clarity, a component of EI, enables individuals to recognize and articulate their emotional states, facilitating proactive strategies to mitigate the impact of stressors on their academic and personal lives.

A significant finding of this study was the negative association between perceived stress and resilience (B = ‐0.48, β = 0.15, t = 7.16, p < 0.001). This aligns with previous research that emphasizes the detrimental impact of stress on resilience. Perceived resilience has been shown to significantly mitigate stress among university students, demonstrating its critical role in reducing the psychological burden of academic pressure (Bukhari et al. 2023). Other studies have highlighted that international students who develop resilience strategies, such as accessing support systems and engaging in group activities, can better manage stress and academic challenges in non‐Western contexts (Ononye et al. 2022). Research has also shown that heightened stress negatively impacts resilience in nursing students, emphasizing the need for interventions targeting emotional regulation and stress management to foster resilience (Hwang and Kim 2023)​.

Furthermore, teacher support has been found to moderate the effects of stress on resilience. Increased support enables students to manage stress better while maintaining higher levels of resilience (Ahmed et al. 2018). The negative correlation between stress and resilience underscores the need for universities to incorporate stress‐management training and resilience‐building initiatives into their curricula. These programs can better equip students to navigate academic and personal challenges, ultimately improving their well‐being and academic performance.

Additionally, this study identified several factors that positively contributed to resilience, including health status, academic year, GPA, and nationality. These findings align with prior research suggesting that students in good health, those with higher academic performance, and those in advanced academic years are more likely to exhibit resilience (Beri and Kumar 2018; Năstasă et al. 2022; Trigueros et al. 2020). Further, Jordanian and Omani students demonstrated higher resilience than their Egyptian counterparts. This finding may reflect cultural or educational system differences, such as variations in family support structures, educational policies, or societal expectations.

This study is among the first to comprehensively explore resilience predictors across three Mediterranean countries, highlighting unique cultural and systemic factors shaping resilience. Furthermore, this study revealed the importance of stress management training and support systems in fostering resilience. Participants who reported access to stress management resources and strong support networks exhibited higher resilience. This finding aligns with evidence suggesting social and institutional support is critical in promoting resilience (Beri and Kumar 2018; Hwang and Kim 2023). Universities can leverage these insights by offering workshops on stress management techniques, creating peer support programs, and fostering a campus culture that prioritizes students’ well‐being.

Interestingly, demographic variables, such as gender, age, part‐time work, extracurricular activities, and field of specialty, were not significant predictors of resilience. This finding is consistent with some studies that failed to identify consistent demographic influences on resilience (Ahmed et al. 2018; Trigueros et al. 2020). The lack of significance in these variables suggests that individual psychological traits, such as emotional intelligence, rather than demographic or situational factors, may more strongly influence resilience. The lack of significance in demographic predictors suggests that psychological traits may influence resilience more than situational factors, aligning with findings from Ahmed et al. (2018). This finding highlights the need for interventions to develop internal resources, such as emotional regulation and coping skills, rather than targeting specific demographic groups.

### 5.1 Implications

This study's findings have important practical implications for educational institutions. To enhance student resilience, universities should prioritize programs that develop emotional intelligence and provide structured support systems. For example, incorporating resilience‐building activities into the curriculum, offering stress management workshops, and creating mentorship programs could help students develop the skills to navigate academic and personal challenges. Universities could integrate these programs into existing orientation sessions for first‐year students, ensuring that emotional intelligence training and stress management resources are accessible from the start of their academic journey. Fostering a supportive academic environment that encourages open communication and access to mental health resources can further bolster student resilience.

### 5.2 Limitations

This study had some limitations. The cross‐sectional design limits the ability to establish causal relationships between variables. Additionally, reliance on self‐reported measures may introduce a response bias, as participants may overreport positive traits or underreport stress levels. Cultural norms regarding the expression of stress and resilience may have influenced self‐reported measures, particularly in countries where discussing mental health remains stigmatized. This potential bias underscores the need for culturally sensitive tools in future research. Future research could address these limitations by adopting a longitudinal design to explore the dynamic nature of resilience development over time. Furthermore, investigating the mediating roles of other psychological constructs, such as optimism, self‐efficacy, and coping styles, could provide a more comprehensive understanding of the mechanisms underlying resilience. Finally, the sample included varying proportions of students from Egypt, Jordan, and Oman, which may reflect underlying cultural and systemic differences in educational environments, social expectations, and student support structures. Although participants were recruited proportionally based on the estimated population of students in each country, these contextual differences may have influenced responses and could limit the generalizability of the findings across broader or different cultural settings.

## 6 Conclusion

This study highlights the interplay between academic resilience, emotional intelligence, and perceived stress among university students in the three Mediterranean countries. Emotional intelligence emerged as a key positive predictor of resilience, enhancing emotional regulation and coping, whereas perceived stress was negatively associated with resilience, underscoring its detrimental effects. Factors such as good health, advanced academic years, and stress management training significantly supported resilience, whereas demographic variables such as gender and field of specialty had no notable impact. These findings emphasize the need for targeted interventions, including emotional intelligence training and stress management programs, to strengthen resilience and help students navigate academic challenges effectively. Future research should explore additional psychological factors and consider longitudinal designs to enhance resilience‐building strategies. By addressing these factors, policymakers and educators can create inclusive, supportive environments that enhance resilience and academic success across diverse cultural settings.

## Author Contributions

**Hanan Abdelrahman**: conceptualization, methodology, formal analysis, supervision, writing–review and editing, writing–original draft. **Mohammad Al Qadire**: conceptualization, methodology, supervision, formal analysis, validation, writing–original draft, writing–review and editing. **Suha Ballout**: writing–review and editing, writing–original draft, data curation. **Mohammad Rababa**: data curation, writing–review and editing, writing–original draft. **Esther Nana Kwaning**: formal analysis, writing–review and editing, resources. **Hamada Zehry**: data curation, supervision, formal analysis, writing–original draft, writing–review and editing.

## Ethics Statement

This study adhered to the principles of the Declaration of Helsinki. Approval was obtained from the Ethics Committee of the College of Nursing at Sultan Qaboos University (Ref. No. CON/NF/2023/14). Additionally, approvals were secured from the ethics committees of Sultan Qaboos University Hospital (REF. NO. SQU‐EC/ 229∖2023 MREC # 3116), and Royal Hospital (REF.NO.RH.21/2021).

## Conflicts of Interest

The authors declare no conflicts of interest.

### Peer Review

The peer review history for this article is available at https://publons.com/publon/10.1002/brb3.70497

## Data Availability

The data that support the findings of this study are available from the corresponding author upon reasonable request. The data are not publicly available due to privacy or ethical restrictions.
